# Challenges in measuring ACGME competencies: considerations for milestones

**DOI:** 10.1186/s12245-018-0198-3

**Published:** 2018-09-28

**Authors:** Prathiba Natesan, Nicholas J. Batley, Rinad Bakhti, Philippe Z. El-Doueihi

**Affiliations:** 1 Department of Educational Psychology, University of North Texas, 1155 Union Circle #311335, Denton, TX 76203 USA; 2 Department of Emergency Medicine, American University of Beirut Medical Center, Hamra, Beirut, Lebanon

## Abstract

**Background:**

Measuring milestones, competencies, and sub-competencies as residents progress through a training program is an essential strategy in Accreditation Council for Graduate Medical Education (ACGME)’s attempts to ensure graduates meet expected professional standards. Previous studies have found, however, that physicians make global ratings often by using a single criterion.

**Methods:**

We use advanced statistical analysis to extend these studies by examining the validity of ACGME International competency measures for an international setting, across emergency medicine (EM) and neurology, and across evaluators. Confirmatory factor analysis (CFA) models were fitted to both EM and neurology data. A single-factor CFA was hypothesized to fit each dataset. This model was modified based on model fit indices. Differences in how different EM physicians perceived the core competencies were tested using a series of measurement invariance tests.

**Results:**

Extremely high alpha reliability coefficients, factor coefficients (> .93), and item correlations indicated multicollinearity, that is, most items being evaluated could essentially replace the underlying construct itself. This was true for both EM and neurology data, as well as all six EM faculty.

**Conclusions:**

Evaluation forms measuring the six core ACGME competencies did not possess adequate validity. Severe multicollinearity exists for the six competencies in this study. ACGME is introducing milestones with 24 sub-competencies. Attempting to measure these as discrete elements, without recognizing the inherent weaknesses in the tools used will likely serve to exacerbate an already flawed strategy. Physicians likely use their “gut feelings” to judge a resident’s overall performance. A better process could be conceived in which this subjectivity is acknowledged, contributing to more meaningful evaluation and feedback.

## Background

Physicians are required to assess trainees in order to monitor their progress. Assessments need to be both valid and efficient to ensure residents receive proper and timely feedback so that corrective measures can be implemented when necessary. The aim of this paper is to demonstrate that commonly used tools which attempt to assess a trainee’s specific skills and attributes via discrete core competencies, or more recently milestones with sub-competencies as prescribed by the ACGME, are actually not measuring these distinct components. Rather, evaluators form gestalt impressions of trainees and translate these “gut feelings” into an overall assessment.

In 2002, the Accreditation Council for Graduate Medical Education (ACGME) introduced six competencies to assess trainees: patient care, medical knowledge, practice-based learning and improvement, interpersonal and communication skills, professionalism, and systems-based practice [1, 2]. More recently, ACGME milestones are being introduced in an attempt to ensure that defined and discrete levels of competence are reached before a resident is deemed fit to practice safely, unsupervised [3]. The six ACGME competencies are currently being expanded such that the milestones will encompass 24 sub-competencies. The reliability and quality of resident assessments, including the objectivity and feasibility of assessing specific clinical abilities as well as non-cognitive attributes, have been questioned [4, 5]. Inaccuracy of reports due to memory loss, selective recall, time constraints, and fatigue, all affect the quality of evaluations [5–7].

Several studies using basic statistical analysis tools have demonstrated that in fact, physicians make global ratings of students often by using a single criterion. Any specific rating on an instrument can predict the overall grade of a trainee because physicians form a general impression of performance rather than judging separate competencies [8]. In one study, a single-item measuring trainee performance had the same reliability as multiple-item scales and reliability only marginally improved with increased items [9]. In a separate study, medical and interpersonal skills emerged as the only two underlying dimensions of the instrument [10]. Physicians’ evaluation of “overall resident competency” has been shown to account for nearly 97% of the variance, providing further evidence of a “halo” effect [2]. Additionally, instruments developed based on the six competencies did not reliably or validly measure the proposed factors [11] or yielded either one or two dimensions that explained the majority of the variance [1, 12–14].

In sum, there is sufficient evidence in the literature for very high correlations between resident evaluation items (Appendix). In statistical terms, this is “multicollinearity.” This indicates that any one item gives the same information as any other item or the rest of the items put together. Clearly, this is a significant psychometric problem. This multicollinearity is also a threat to validity because the items are clearly not actually measuring the six AGCME competencies they aim to assess.

Another issue with trainee evaluations is that most of these criteria are generally measured using categorical scales which yield ordinal data. For instance, one of the ACGME global evaluation forms recommended on their website (http://www.acgme.org/Portals/0/430_RadOnc_GlobalRev.pdf) attempts to measure the six core competencies on a 9-point scale ranging from 1 to 3 representing unsatisfactory, 4–6 representing satisfactory, and 7–9 representing superior. Another example from the University of Maryland Medical center uses a 4-point scale with 0 representing not applicable, 1—below expectations, 2—meets expectations, and 3—exceeds expectations. Using categorical scales to measure complex phenomena poses the question as to whether evaluators can reliably convert a continuous variable such as core competency and convert it to a 3- or 9-point scale which is ordinal. Moreover, using such categorical scales to measure competencies requires additional precautions that need to be taken while conducting statistical analysis, because considering categorical data as continuous can lead to erroneous conclusions.

The recent milestone approach focuses on an outcomes-based process by including explicit accomplishments or behaviors that become progressively more advanced during residency training. Milestones aim to introduce more specific competencies tailored to every specialty and incorporate objective measures from multiple assessment tools [15]. Importantly, the developed milestones are derived mainly from previous core ACGME competencies expanded to 24 sub-competencies. The question of whether the expansion from six core competencies to 24 sub-competencies would yield information that is more useful is questionable. As ACGME is in the process of developing its milestones approach, a methodologically sophisticated study that thoroughly examines the issues in measuring the basic six competencies is necessary. Our study does this and as such can help inform future directions for milestone development.

Although studies have investigated the six core competencies, there are several gaps in the existing literature [8, 10]. First, most of these studies used univariate analysis or exploratory factor analysis (EFA). Core competency is a complex multivariate construct and employing univariate analyses reduces the complexity of this construct and yields an incomplete picture of the results. EFA cannot confirm that our theory is adequately represented by our data. This requires confirmatory factor analysis (CFA). Second, most of these studies do not apply corrections for their categorical scale of measurement (ordinal) and the possible non-normality that accompanies ordinal data. Only one study assessed their data for possible non-normality [13], and one study reported transforming ordinal data into interval data [12]; the procedure for this transformation was not reported. Ignoring the ordinal nature of the data can lead to severely inaccurate estimates [16, 17]. A simple example would be to consider ranks, which are categorical (ordinal data) versus scores which are continuous (interval data) for three subjects. Let us say that their scores are 100, 95, and 94.5 which means their ranks are 1, 2, and 3, respectively. What rank ignores is the fact that the distance between 100 and 95 is larger than the distance between 95 and 94.5. Thus, even computing averages for categorical data is meaningless, let alone conducting advanced statistical analysis on it. Third, it is not known whether all evaluators convert a continuous variable such as rating on a core competency identically to a categorical variable on a 6- or 8-point scale. Finally, it is not known whether the evaluations of these competencies are consistent across different specialties of medicine, in a non-US clinical setting, and across evaluators (physicians). In order to ascertain this last point, it is necessary to conduct measurement invariance testing.

Measurement invariance is a series of tests conducted to establish if factor scores such as core competency scores are measured across evaluators (or groups) on the same metric so they are comparable. The first model, configural invariance model, tests if the same model structure exists across all groups. The second model, metric invariance, tests if the factor coefficients, that is the relationship between the items and the underlying factor are identical across evaluators. The third model, scalar invariance model, tests if the means of the items (in addition to the factor coefficients) are identical across the groups. Error variance invariance model, the final model, checks if the error variances of the items are identical across groups. Lack of metric, scalar, or error variance invariance indicates that one evaluator is stricter than another evaluator or perceives the items differently from each other. That is, for the same student, two evaluators will give different scores on the competency items if there is lack of invariance. This indicates potential bias. Therefore, comparing core competency scores across these evaluators would not be fair. When there is measurement invariance (that is, lack of variation in how things are measured across groups/evaluators), there is construct validity because the construct, core competency is identically defined across evaluators.

This study examines three types of validity for resident evaluations at a tertiary academic medical care center in Beirut, Lebanon. We test (a) convergent validity by examining the hypothesized single-factor structure for emergency medicine (EM) resident evaluations, that is, we test if the six core competency items all are uniquely and significantly indicating different aspects of the underlying construct, core competency; (b) replicability across departments by examining if the single-factor structure also holds true for neurology resident evaluations; and (c) construct validity by examining the consistency of this factor structure across EM evaluators, that is, we test if this indication by items or the relationship between the underlying construct and the individual items is independent of the evaluator. This would mean that all items are perceived and rated identically by all evaluators. Our study is significant because it comes at a crucial time when ACGME is revamping core competencies and moving to a milestones approach.

## Methods

This retrospective study has been approved by the Institutional Review Board at the American University of Beirut. The medical center resides in the heart of Beirut, Lebanon, and hosts residency programs in all major specialties. The residency programs are Accreditation Council for Graduate Medical Education International (ACGME-I) accredited and fulfill their requirements to provide evaluation and feedback on a scheduled and regular basis. In the EM department, the six core competency items were measured on a 6-point categorical scale whereas in the neurology department, they were measured on an 8-point categorical scale. The 6-point scale in the EM department ranged from 1 to 2 representing unsatisfactory, 3–4 representing satisfactory, and 5–6 representing superior, while the 8-point scale in the neurology department was measured on a sliding scale. Because of the 8-point categorical scale, psychometric literature permits us to consider the neurology data as intervally scaled. However, EM data has only six categories and was therefore considered ordinally scaled for the purpose of the analysis. Ordinal alphas were used to examine the internal consistency of the EM data because computing regular alphas for data with fewer than seven categories can produce inaccurate alpha estimates [16]. Regular coefficient alphas were used to examine internal consistency of the scores for neurology data. Fifty-nine evaluators evaluated 58 residents (both EM and non-EM) in the EM department once every 3 months. This resulted in 531 evaluations. For neurology, 14 evaluators evaluated 13 residents once every 2–4 weeks. This resulted in 93 evaluations.

**Fig. 1:**
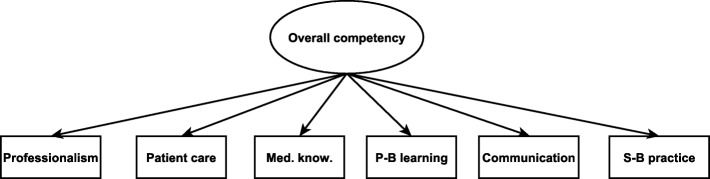
The confirmatory factor analytic model showing the relationship between overall competency and the core competencies

Confirmatory factor analyses (CFA, Fig. 1) were used to examine the structure of the instrument for each department. The CFA model theorizes that the six core competency items uniquely and significantly indicated the underlying construct called core competency. Through model fitting, we investigated if this model was reflected by the data. When our model-based statistics are close to the sample-based statistics, we can conclude that we have good model fit. Model fit is determined by cut-off scores on fit indices as prescribed by the literature. Measurement invariance (MI) across evaluators was tested to examine if each EM evaluator perceived the constructs identically. Presence of MI would support construct validity by indicating that the constructs are defined identically across evaluators or groups [18]. Weighted least squares means and variances adjusted (WLSMV) [19] estimates were used because of the ordinal and non-normal nature of the EM data. Good model fit was indicated when comparative fit index(CFI) > 0.95, and root mean square of approximation (RMSEA) and standardized root mean square residual (SRMR) < 0.08 [20]. Factor coefficients greater than 0.95 indicated that the construct shared more than 90% of the variance with the item, hence, multicollinearity. That is, the item can replace the entire underlying construct or the vice versa. Therefore, deleting either one of these will not affect the amount of information provided by the data. Lavaan package in R was used to fit the models [21].

## Results

Ordinal coefficient alpha for EM was 0.93 and ranged from 0.86 to 0.985 between evaluators. Coefficient alpha for neurology was 0.95. Although alpha values greater than 0.8 are considered desirable, very high alpha values may indicate high-shared variance (i.e., multicollinearity). Unlike other studies, only 6.65% of our data were straight-lined. Straight lining happens when the participants select the same answer choice for all items. The single factor model with core competency as the single underlying factor indicating all six items fit the EM data well but had severe multicollinearity. This is because the unstandardized factor coefficients were 0.98 or higher for all items as shown in Table 1 [22] (${\chi}_{\mathrm{scaled}}^2=28.062,p=0.001,\mathrm{CFI}=0.95,\mathrm{RMSEA}=.064\left[.038,.091\right],\mathrm{SRMR}=.02$). Similarly, all factor coefficients were 0.95 or higher for the neurology data also indicating severe multicollinearity (*χ*^2^ = 101.94, *p* < .005, CFI = 0.819, RMSEA = .329[.271, .390], SRMR = .08). This model fits the data poorly.

**Table 1:** Unstandardized factor coefficients of the six core competencies for EM and neurology resident evaluations

Core competencies	EM	Neurology
Medical knowledge	1	1.01
Patient care	1.01	1.00
Systems-based practice	1.33	0.99
Practice-based learning	0.98	0.99
Professionalism	1.24	1.18
Interpersonal and communication skills	1.01	1

Next, we fitted a multi-group CFA (MCFA) model by evaluator for EM data to identify if some evaluators distinguished between the six competencies. The model could be tested on only six core EM faculty evaluators who evaluated between 36 and 58 residents. This is because we needed sufficient sample size to conduct this analysis. The CFA model fit only three of the six evaluators (i.e., evaluators 11, 20, and 41). The unstandardized factor coefficients were 0.94 or higher for evaluators 11 and 41, whereas they ranged from 0.587 to 1 for evaluator 20. Next, a multi-group CFA model with factor coefficients fixed to be the same between the 3 evaluators had negative error variance indicating bad model fit. Only the metric invariance model between the two evaluators passed Chen’s [23] cutoff criteria for measurement invariance (Δ*CFI* ≤  − .005, ΔRMSEA ≥ .01, ΔSRMR ≥ .025). Model with scalar invariance across the two evaluators did not pass the criteria (Table 2). However, the metric invariance model also had extremely high factor coefficients.

**Table 2:** Measurement invariance tests across the two EM evaluators

Model	${\chi}_{\mathrm{scaled}}^2$	*df*	*p*	CFI	RMSEA	SRMR	ΔCFI	ΔRMSEA	ΔSRMR
Configural	18.51	18	0.422	0.997	0.025	0.006			
Metric	20.39	23	0.618	1.000	0.000	0.011	− 0.003	0.025	− 0.005
Scalar	29.72	28	0.376	0.990	0.036	0.017	0.010	− 0.036	− 0.006

Δ = previous model-current model

## Discussion

The objective of the assessments analyzed in this study was to evaluate residents’ six core competencies. The results, however, demonstrate that any single item essentially can replace every other item. CFA models for both EM and neurology fit the data poorly. The high factor coefficients indicate that evaluators do not distinguish between the competencies. This means the data does not support the model where all items are uniquely and significantly indicating a single underlying construct. Only evaluators 11 and 41 perceived the items to have the same relationship to the underlying construct. However, the factor coefficients were very high for this model. Therefore, even though these two evaluators perceived some aspects of the six items identically, there is no support for the items being perceived as unique from each other, both at the group level and at the individual evaluator level.

The results are the same irrespective of the scale of measurement, the cultural setting, the department, or the evaluator. In conclusion, this assessment and those like it may be useful only for rating the overall competence of residents but presents little information on their specific strengths and weaknesses in the six competencies. When presented with the instrument, evaluators have possibly formed a global perspective of the residents, which they then apply to the specific competencies. This finding is in line with previous studies that suggest that a global impression by evaluators guides their responses on individual competencies.

More than 97% of specialty programs in the USA employ assessment forms based on the ACGME milestones/competencies[6]. Since the introduction of the ACGME competencies, residency programs have likely increased the number of items in resident evaluations to reflect these suggestions [2]. However, ACGME suggestions may impose a certain artificiality to resident assessments that is not intuitive to evaluators [10]. For example, distinguishing professionalism and interpersonal and communication skills in the mind of evaluators can be challenging. This inability to distinguish between the competencies may stem from an implicit overlap between the concepts. Another explanation is that in addition to the halo effect, central tendency, which results from assessing residents in a restricted and narrow range (usually highly positive) may also be biasing the data [2].

Incorporating a more qualitative approach and assessments that are less standardized and structured can have great utility [24, 25]. Competency-based medical assessment (CBME) is multifaceted in nature and would benefit from involving qualitative measures, especially with competencies that may be difficult to quantify, with some studies encouraging the use of narrative descriptions [24].

Some objective data about resident performance can be gathered such as the number of patients who return to the emergency department within 72 h, the load of patients each resident sees, their turnaround times, and lab utilization. When we observe a resident at work, however, we also form subjective, qualitative feelings about their competence. By attempting to convert the combination of those objective data and feelings into numbers on a form, we turn this complex and nuanced assessment into the comfort of numeric data, which this paper clearly shows, is a very challenging task and provides an incomplete picture. So far, there has been no reasonable alternative to attempting to measure the core competencies on a categorical scale.

### Limitations

Our study has some limitations. First, although the scales include anchors to help raters match numbers with performance, faculty members received no direct training on how to interpret and make use of the scale. Second, the number of faculty members is small compared to most US programs. Nonetheless, it still confirms the findings from some US-based studies that most evaluators evaluate residents in a global manner and do not discriminate between various core competencies.

## Conclusions

The move towards ACGME milestones with 24 sub-competencies makes the task even more challenging and will most likely exacerbate the severe multicollinearity seen in this and previous studies. A better approach might be to recognize and embrace the part of the assessment process that is subjective. All the time a supervising physician spends with a resident can be viewed as microscopically parsed moments; each one contributing to the impression the resident is making in the evaluators mind. We do not make assessments for six competencies once a quarter, we form our opinions every second we interact with and observe the resident. As Georges-Pierre Seurat created his “Bathers at Asnières” masterpiece using a multitude of infinitesimally discreet points, so our assessment of a resident’s performance is an overall picture formed from every moment of every interaction. If we acknowledge this and recognize the importance and validity of time spent forming “gut feelings” [26], we may more comfortably include these “feelings” in our resident assessments. These necessarily subjective assessments could then be discussed with the resident, perhaps using the ACGME milestones and sub-competencies as a framework, to form a much richer and meaningful form of assessment and feedback while relieving busy physicians the burdensome task of filling out evaluation forms that are not measuring what they are intended to measure.

## Appendix

**Table 3:** EM resident evaluation

Medical knowledge: demonstrates basic science and clinical knowledge	
Patient care: overall ability to provide patient care	
Systems-based practice: ability to use systems and resources appropriately	
Practice-based learning: ability to learn from experience, follows up on patients	
Professionalism: motivation, integrity, dependability, responsibility, respect for others	
Interpersonal and communication skills: works as part of a team, shows respect, is able to listen, communicates ideas clearly	

## Abbreviations

ACGME: Accreditation Council for Graduate Medical Education
ACGME-I: Accreditation Council for Graduate Medical Education International
CBME: Competency-based medical assessment
CFA: Confirmatory factor analysis
CFI: Comparative fit index
EFA: Exploratory factor analysis
EM: Emergency medicine
MCFA: Multigroup confirmatory factor analysis
MI: Measurement invariance
RMSEA: Root mean square of approximation
SRMR: Standardized root mean square residual
WLSMV: Weighted least squares means and variances adjusted
